# Metastatic mixed acinar-endocrine carcinoma of the pancreas treated with a multidisciplinary approach: a case report

**DOI:** 10.1186/s40792-017-0326-y

**Published:** 2017-03-30

**Authors:** Takeo Hara, Yoshiyuki Fujiwara, Hidenori Takahashi, Keijiro Sugimura, Jeong-Ho Moon, Takeshi Omori, Norikatsu Miyoshi, Akira Tomokuni, Hirofumi Akita, Shogo Kobayashi, Masayoshi Yasui, Hiroshi Miyata, Masayuki Ohue, Masato Sakon, Yasuhiko Tomita, Masahiko Yano

**Affiliations:** 1 Department of Surgery, Osaka Medical Center for Cancer and Cardiovascular Diseases, 1-3-3 Nakamichi, Higashinari-ku, Osaka City, Osaka 537-8511 Japan; 2 Department of Diagnostic Pathology, Osaka Medical Center for Cancer and Cardiovascular Diseases, 1-3-3 Nakamichi, Higashinari-ku, Osaka City, Osaka 537-8511 Japan

**Keywords:** Mixed acinar-endocrine carcinoma, Pancreatic neoplasm, Metastatic gastric tumour

## Abstract

**Background:**

Pancreatic neoplasms are usually characterized by ductal, acinar, or endocrine differentiation. Mixed exocrine and endocrine pancreatic tumours are extremely rare. Here, we report a case of pancreatic mixed acinar-endocrine carcinoma (MAEC) with multiple synchronous liver metastases that were treated with surgery and transcatheter arterial chemoembolization (TACE) that later recurred in the stomach.

**Case presentation:**

A 45-year-old female with severe anaemia was referred to our hospital. Computed tomography (CT) demonstrated a hypervascular tumour, 17 cm in diameter, that was in the tail of the pancreas. In addition, there were multiple hypervascular tumours in the liver. She underwent a distal pancreatectomy with splenectomy after the liver metastases were treated with TACE. Pathology confirmed that the pancreatic tumour was MAEC. After 4.5 years, a follow-up CT showed a hypervascular tumour at the upper part of the stomach. Gastric endoscopy showed a big tumefactive lesion with surface irregularities, gastric erosion, and multiple dilated vessels in the fornix and greater curvature of the stomach. She underwent a proximal gastrectomy and survived 7 years and 2 months after the start of the treatment.

**Conclusions:**

This is the first report of a metastatic stomach tumour from pancreatic MAEC, which was successfully treated with a multidisciplinary approach. Additionally, we review the literature and discuss the treatment of MAEC.

## Background

Mixed acinar-endocrine carcinoma (MAEC) is a rare neoplasm that is mainly observed in the pancreas. According to the WHO classification, MAEC is defined as a variant of acinar cell carcinoma and should have an endocrine component in more than 30% of tumour cells [1, 2]. However, the biological behaviours, treatment strategies, and overall prognosis for patients with MAEC are still unclear. Here, we report the case of a patient with pancreatic MAEC with multiple liver metastases who underwent distal pancreatectomy and catheter-directed treatment to the liver. She subsequently developed a metastatic tumour to the stomach 4.5 years later. She was successfully treated with a proximal gastrectomy for the metastatic stomach tumour. This is the first report of a metastatic stomach tumour from pancreatic MAEC that was successfully treated with a multidisciplinary approach. To date, 12 cases of pancreatic MAEC that met criteria by the WHO classification have been reported in the English literature, which we identified by a PubMed search [3–10]. We review the literature and discuss the treatment for MAEC.

## Case presentation

**Fig. 1:**
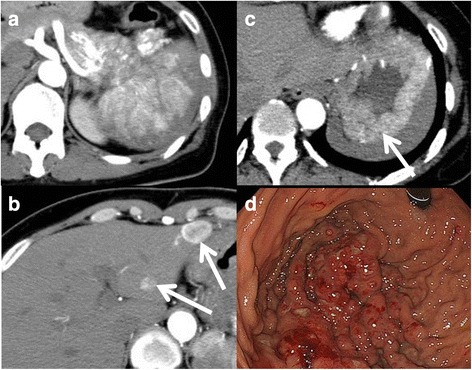
Abdominal contrast-enhanced CT findings and upper endoscopy findings. CT showing a hypervascular tumour in the tail of the pancreas, infiltrating the spleen and the splenic vein (**a**). Multiple hypervascular tumours in the liver (**b**). Hypervascular tumour in the upper part of the stomach (**c**). Upper endoscopy showing a big tumefactive lesion in the fornix and greater curvature (**d**)

The patient was a 45-year-old female who presented with severe anaemia (Hb 4.7 g/dl) on a laboratory test performed by a local doctor. Gastroscopy revealed gastric varices with blood oozing and an extrinsic compression in the gastric wall. Her anaemia was improved by transfusion and oral iron preparation. On physical examination, she had a soft, non-tender abdomen without any palpable masses. The laboratory data showed the following: Hb 11.8 g/dl (reference range, 12–15 g/dl), aspartate aminotransferase (AST) 12 U/l (13–33 U/l), alanine transaminase (ALT) 7 U/l (6–27 U/l), total bilirubin 0.2 mg/dl (0.2–1.2 mg/dl), and total amylase 49 IU/l (33–120 IU/l). Tumour marker levels were as followed: carcinoembryonic antigen (CEA) 2.5 ng/ml (<5 ng/ml), cancer antigen 19-9 (CA19-9) 0 U/ml (0–36 U/ml), DUPAN-II 215 U/ml (<150 U/ml), and elastase 281 ng/dl (28–254 ng/dl). She had no specific medical or family history of this disease. Computed tomography (CT) demonstrated a hypervascular tumour that was 17 cm in the tail of the pancreas, infiltrating the spleen and the splenic vein (Fig. 1a). In addition, there were multiple hypervascular tumours in the liver (Fig. 1b). CT-guided core needle biopsy of a liver tumour was performed, and pathological examination revealed well-differentiated endocrine carcinoma originating from the pancreas. She was diagnosed with pancreatic endocrine carcinoma with multiple liver metastases. First, transcatheter arterial chemoembolization (TACE) with epirubicin 50 mg + Lipiodol 7 ml + GS cube was performed to treat the liver metastases. After confirmation that the liver metastases were under control, she underwent distal pancreatectomy and regional lymph node dissection with splenectomy. Pathological examination showed that the pancreatic tumour was MAEC. She was followed without any treatment including TACE after surgery. Four years and 5 months later, routine haematology labs showed progressive anaemia, and CT showed a hypervascular tumour located in the upper part of the stomach (Fig. 1c). The liver metastases were stable. Gastric endoscopy showed a large tumefactive lesion with surface irregularities, gastric erosion and multiple dilated vessels in the fornix and greater curvature of the stomach (Fig. 1d). Biopsies performed during endoscopy confirmed metastatic gastric tumour from MAEC. Therefore, she underwent a proximal gastrectomy with cholecystectomy for gallstone disease with double-tract reconstruction. The patient survived for 2 years and 7 months after gastrectomy, although TACE was performed for the recurred liver metastasis twice.

### Macroscopic and microscopic examination

**Fig. 2:**
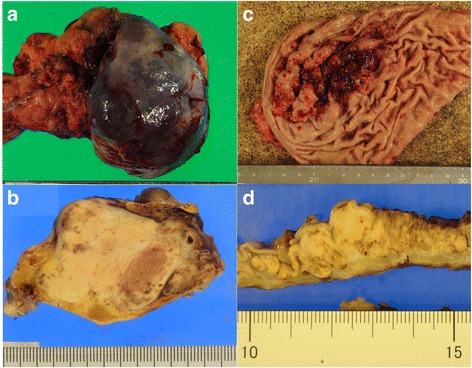
Photograph of Surgical specimen. Pancreas tumour measuring 11 cm, which was white in colour and well demarcated, had expansive lobular growth, and was located in the tail of the pancreas, infiltrating the spleen and the splenic vein (**a**, **b**). Gastric tumour measured 9 cm. Tumour was white in colour with a distinct border and had expansive growth under the submucosa (**c**, **d**)

The resected pancreas tumour measured 11 cm and was in the tail of the pancreas, infiltrating the spleen and the splenic vein (Fig. 2a, b). Tumour thrombus was found from the main tumour to the splenic vein. However, no lymphatic invasion and no regional lymph node metastasis were identified. The resected gastric tumour measured 10 cm in the fornix and greater curvature (Fig. 2c, d).

**Fig. 3:**
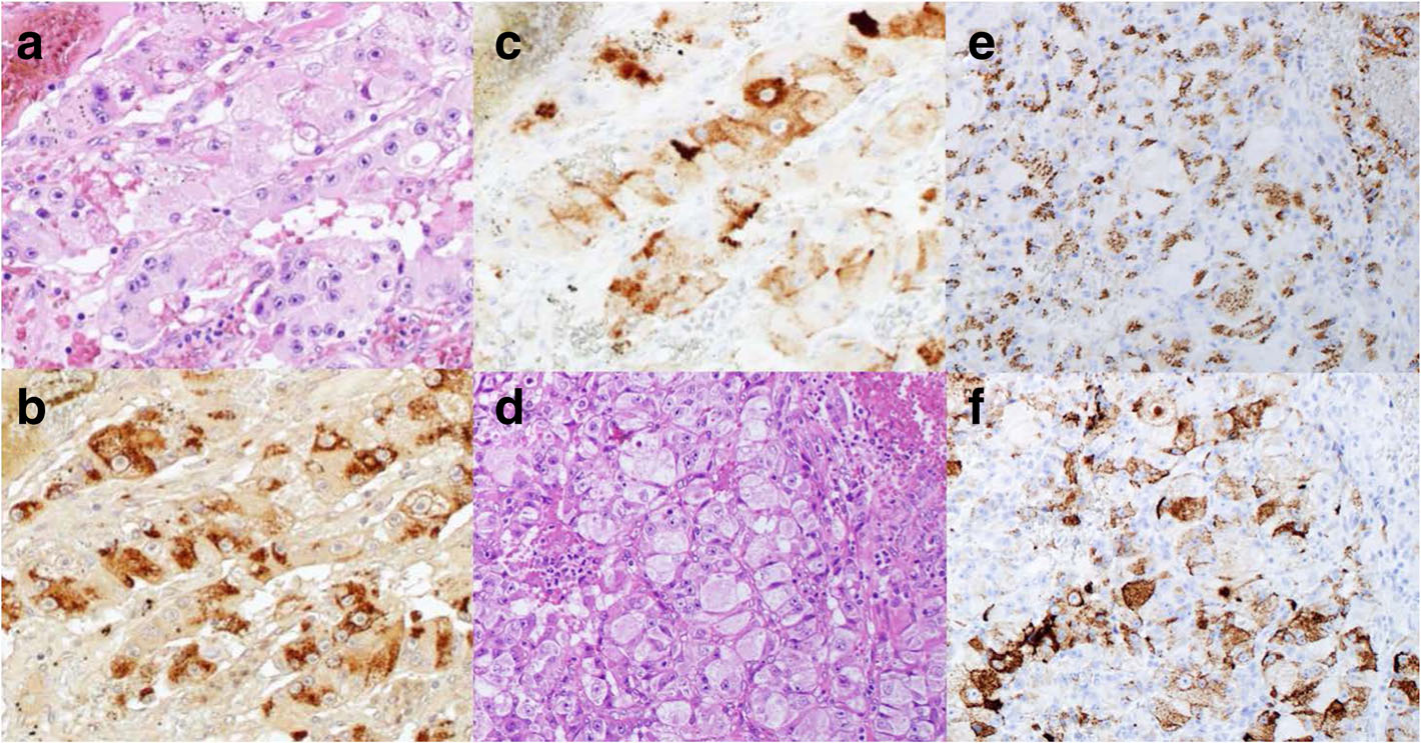
Histopathological features of the mixed acinar-endocrine carcinoma. Haematoxylin and eosin image of the pancreas tumour, 100× (**a**). Immunostaining of the pancreas tumour for trypsin 100× (**b**) and chromogranin A 100× (**c**). Haematoxylin and eosin image of the gastric tumour, 50× (**d**). Immunostaining of the gastric tumour for trypsin 50× (**e**) and chromogranin A 50× (**f**)

Histology confirmed a cellular neoplasm originating from the pancreatic parenchyma. Two distinct populations of neoplastic cells were present. Many tumour cells grew in solid nests and included acinar and glandular structures (Fig. 3a). Immunohistochemistry (IHC) studies demonstrated strong co-expression of acinar and endocrine markers in all the tumour cells (Fig. 3b, c). Most of the tumour was positive for trypsin, an acinar cell marker (more than 90% of the tumour), and many cells were positive for chromogranin A, an endocrine marker (40–50% of the tumour). Histology confirmed a cellular neoplasm originating from the gastric tumour that showed acinar arrangement or glandular formation with eosinophilic secretory material (Fig. 3d). Immunohistochemical staining of metastatic gastric tumour was positive for both trypsin and chromogranin A in more than 40% of the tumour (Fig. 3e, f). Therefore, the patient was diagnosed with MAEC metastatic to the stomach.

### Discussion

The pancreas is well known to be composed of two entirely different components, the exocrine and the endocrine pancreas. The exocrine pancreas is composed mainly of duct cells with some contribution from acinar cells, whereas the endocrine pancreas consists of islet cells. One of the most important features of pancreatic neoplasms is the type of differentiation of the cancer cells, which determines the classification of the tumour. Most pancreatic epithelial neoplasms recapitulate to some degree one or more of the normal epithelial cell types of the pancreas: ductal (75–90%), acinar (1–2%), and endocrine (3–7%) [11]. Some pancreatic neoplasms have significant components from more than one line of differentiation and are classified as mixed acinar-endocrine carcinomas (MAEC), mixed acinar-ductal carcinomas, mixed ductal-endocrine carcinomas, or mixed acinar-endocrine-ductal carcinomas [2]. By arbitrary definition, each component must comprise at least 25% of the neoplasm [3, 12]. Only 21 cases that fit this strict definition have been reported in the English literature.

Table 1 summarizes the 21 cases of pancreatic MAEC that have both acinar and endocrine components in more than 25% of tumour cells by immunohistochemistry reported in the English literature, excluding the cases diagnosed only by biopsy [1, 3–10, 13–15]. In these reports, 16 of the 21 patients with confirmed MAEC of the pancreas were male (76%). The median age was 65 years (range 45–89 years), and our case involved the youngest patient. MAECs were most commonly located in the head of the pancreas. The median tumour size of MAECs was 7.3 cm. Nineteen patients underwent surgical resection, 1 patient underwent bypass surgery (gastrojejunostomy), and the remaining 1 patient received chemotherapy only. Out of the 19 patients who underwent surgical resection, 2 received preoperative chemotherapy, 16 patients underwent curative surgery, and 6 patients received adjuvant chemotherapy. Pathological examination of the surgical or autopsy specimens showed that 12 tumours had predominately solid morphology, 6 had predominately acinar morphology, and the other 3 had both morphologies in equal parts. Immunohistochemistry revealed that 6 tumours had predominately endocrine components, 10 had predominately acinar components, and the other 5 contained equal amounts of both components or showed a co-expression pattern. Eight reports including the current case mentioned on the status of regional lymph node metastases and 4 (50%) out of 8 patients had lymph node metastases [1, 3, 5, 6, 10, 13, 14]. Ten (48%) out of all 21 patients with confirmed MAEC of pancreas had distant metastases. One patient had metastases in multiple organs; however, the details were not provided [5]. The remaining 9 patients had liver metastases. Additional metastasis was observed in kidney, lung, and stomach in the current case.

**Table 1:** Summarizing statistics of cases of mixed acinar-endocrine pancreatic carcinoma (21 cases including the present case)

Characteristics	Frequency (21 cases)
Sex	
Male	16 (76%)
Female	5 (24%)
Age (years)	65 (45–89)
Morphology (predominately)	
Acinar	6 (29%)
Solid	12 (57%)
Both	3 (14%)
Immunohistochemistry (predominately)	
Acinar	10 (48%)
Endocrine	6 (29%)
Both	5 (24%)
Size (cm)	7.3 (1.5–16)
Location	
Head	12 (57%)
Body	2 (10%)
Tail	7 (33%)
Treatment	
Surgical resection	11 (52%)
Surgical resection + chemotherapy or other treatments	8 (38%)
Bypass	1 (5%)
Chemotherapy	1 (5%)
Surgical curability	
R0	16 (76%)
Others	5 (24%)
Metastasis	There is some overlapping
Liver	10 (48%)
Kidney	1 (5%)
Lung	1 (5%)
Stomach	1 (5%)
Multiple organs	1 (5%)

Pancreatic MAEC is very rare with only 21 cases reported in the English literature, as shown in Table 1. Therefore, precise clinical features of MAEC are not well understood and no standard treatment has been established. According to previous reports, the prognosis of MAEC was poor similar to that of acinar cell carcinoma [3, 7]. The prognosis of 16 cases who underwent surgical resection for pancreatic MAEC was analysed; 1-year overall survival rate was 80% and 3-year survival rate was 60% by Kaplan-Meier method [1, 3, 5–7, 9, 10, 14]. We performed a pancreatectomy for pancreas MAEC after arterial catheter embolization for multiple liver metastases. Furthermore, a proximal gastrectomy was also performed for a stomach recurrence, which occurred 4.5 years after the first surgery. The patient survived more than 7 years after she was first diagnosed with pancreatic MAEC and survived for longest term after surgical resection in comparison with previous reports. Because there are no promising anti-tumour therapies, such as chemotherapy or chemoradiation, surgical resection is the first-line therapy for resectable cases of MAEC. Local therapy, such as catheter-directed embolization, is also promising for multiple liver metastases, which is the most common site of metastasis from pancreatic MAEC.

## Conclusions

In conclusion, we presented here a very rare case of metastatic gastric tumour from pancreatic MAEC with multiple liver metastasis. This is the first report of metastasis to the stoma*c*h from pancreatic MAEC. The stomach should be considered a target site of metastasis during the treatment of pancreatic MAEC. A multidisciplinary approach with surgery and TACE for liver metastases might be a promising therapeutic approach for patients with advanced cases of MAEC.

## Abbreviations

CT: Computed tomography
IHC: Immunohistochemistry
MAEC: Mixed acinar-endocrine carcinoma
TACE: Transcatheter arterial chemoembolization
